# Ferroptosis Enhances T Lymphocyte Infiltration into Glioblastoma Spheroids

**DOI:** 10.3390/antiox14111373

**Published:** 2025-11-19

**Authors:** Anna Schwantes, Yara Shadid, Vanesa Maria Guerrero Ruiz, Blerina Aliraj, Anja Wickert, Megan A. Palmer, Sofie P. Meyer, Andreas Weigert, Bernhard Brüne, Dominik C. Fuhrmann

**Affiliations:** 1 Institute of Biochemistry I, Faculty of Medicine, Goethe University Frankfurt, 60629 Frankfurt, Germany; 2 Frankfurt Cancer Institute, Goethe University Frankfurt, 60629 Frankfurt, Germany

**Keywords:** T cells, immune cell infiltration, ATP, lipid peroxidation

## Abstract

Glioblastoma is one of the most aggressive and therapeutically challenging brain tumors. It is characterized by a highly immunosuppressive tumor microenvironment and poor prognosis, requiring novel treatment strategies. Along this line, ferroptosis has been proposed. To study the impact of ferroptosis on glioblastoma cells and immune cell infiltration, we established a spheroid model using LN229 glioblastoma cells and verified ferroptosis by measuring lipid peroxidation and RNA expression of ferroptosis-related genes. We then co-cultured spheroids with human peripheral blood mononuclear cells to follow the infiltration of distinct immune cell subsets by flow cytometry and immunohistochemistry. T lymphocyte infiltration into ferroptotic spheroids compared to control spheroids became apparent with the notion that ferroptotic cells attracted T cells more efficiently compared to apoptotic or necrotic cells. Mechanistically, ferroptotic glioblastoma spheroids released high amounts of ATP, which caused T cell attraction, while ATP deprivation reduced this effect. Ferroptosis appears to be an interesting therapy approach but might need co-treatments to ensure proper T cell activation.

## 1. Introduction

Ferroptosis is a recently discovered distinct form of cell death. It is characterized by its iron-dependency, reactive oxygen species production, and increased lipid peroxide formation [1]. Ferroptosis is defined as a regulated form of cell death occurring in response to oxidative perturbations, which is inhibited by iron chelators and lipophilic antioxidants [2]. Oxidative degradation of membrane-bound polyunsaturated fatty acids causes membrane damage, loss of membrane integrity, and consequently cell death [3]. Inducing ferroptosis in cancer cells is a promising therapeutic approach, especially for drug-resistant tumors, as it is distinct from other types of cell death, such as apoptosis and necroptosis [1].

Migration of immune cells towards dying cells is vital for maintaining tissue homeostasis, initiating immune responses, and tissue regeneration [4,5,6]. Dying cells release various signals, depending on the type of cell death, to orchestrate chemotaxis [7]. Thereby, distinct immune cells are recruited to sites of cell death dependent on the signal and their spatio-temporal release. Immunogenic apoptosis is linked to the active release of chemoattractants, such as chemokines and extracellular nucleotides [8,9,10,11,12], whereas necrotic cell death is associated with an uncontrolled passive release of cellular constituents in danger-associated molecular patterns (DAMPs), as it is normally contained within organelles or cells and/or present in the extracellular space at low concentrations, only. Once released, it activates the innate immune system [10]. Thereby, DAMPs are recognized by pattern recognition receptors on leukocytes, directing them to the site of their origin [10,11,13]. Mechanisms how ferroptotic cells attract or influence immune cell migration are currently under debate and highly dependent on the individual context, i.e., the cell type undergoing ferroptosis and distinct tumor microenvironment [14,15,16].

Glioblastoma is an aggressive and highly therapy-resistant brain tumor, accounting for one-third of all brain or central nervous system tumors and more than 50% of all malignant brain tumors in the United States [17,18]. Their treatment is a formidable challenge, and despite advancements, the prognosis remains dismal, with a median survival of 15 months and a poor five-year survival rate below 10% [18,19]. Glioblastomas belong to the group of cold tumors, characterized by an immunosuppressive microenvironment and a low number of infiltrated immune cells, especially T lymphocytes, contributing to immune evasion and the poor treatment response [20,21,22]. Furthermore, the lack of immune cells renders many immunotherapies, such as immune checkpoint inhibitors, ineffective [20].

Since glioblastoma treatment is currently limited, the induction of ferroptosis holds great potential for glioblastoma therapy. Within this context we aimed to explore ferroptosis in glioblastoma spheroids and concurrent immune cell infiltration.

## 2. Materials and Methods

### 2.1. Cell Culture LN229 and Spheroid Generation

Glioblastoma cells of the human LN229 cell line were purchased from ATCC (Manassas, VA, USA) and cultured in Gibco (Billings, MT, USA) RPMI media containing 10% fetal calf serum and 1% penicillin/streptomycin at 37 °C and 5% CO_2_. For spheroid generation, cells were seeded into 96-well BIOFLOAT™ spheroid plates (83.3925.400, Sarstedt, Nümbrecht, Germany) at 0.05 × 10^6^ cells per well. Media was changed after 1 and 4 days. Spheroids were used for experiments from day 4 to day 6. If spheroids were pooled, they were treated identically in separate wells before harvesting.

### 2.2. PD-L1 Knock-Down Cells

A stable gene silencing of PD-L1 was generated in LN229 glioblastoma cells through RNA interference, using TRC lentiviral shRNA against PD-L1 (RHS4533-EG29126, Horizon Discovery, Cambridge, UK) and a non-targeting control (RHS6848, Horizon Discovery).

### 2.3. Isolation of Peripheral Blood Mononuclear Cells (PBMCs)

Human peripheral blood mononuclear cells (PBMCs) were isolated from Buffy coats using Leucosep tubes (Greiner bio-one, Frickenhausen, Germany) and Biocoll Separating Solution (Biochrom, Berlin, Germany). Cells were washed three times with PBS/EDTA. Erythrocyte depletion was performed by hypotonic lysis. Briefly, cell pellets were resuspended in 12 mL ice-cold water, and then 4 mL 0.6 M potassium chloride solution (104936, Merck, Darmstadt, Germany) were added for isotonic recovery followed by PBS/EDTA. After centrifugation cells were resuspended in media and counted on a TC20 automated cell counter (Bio-Rad Laboratories, Hercules, CA, USA) using trypan blue staining (Bio-Rad Laboratories) to exclude dead cells. PBMCs were cultured in RPMI media supplemented with 3% human plasma and 1% penicillin/streptomycin.

### 2.4. Treatments

To ensure the viability of PBMCs in co-culture, LN229 cells and spheroids were cultured in Gibco RPMI media supplemented with 3% human plasma and 1% penicillin/streptomycin during the experiments. RSL3 (SML2234, Sigma-Aldrich, St. Louis, MO, USA) and liproxstatin-1 (17730, Cayman Chemicals, Ann Arbor, MI, USA) were used at a final concentration of 1 µM. For co-culture experiments, spheroids were treated with 1 µM RSL3 ± 1 µM liproxstatin-1. After 6 h PBMCs were added, which resulted in a final concentration of 0.5 µM RSL3 and liproxstatin-1. Apoptosis was induced by UV-light for migration assays (2D culture) with the energy set to 400 mJ/cm^2^ in a UV crosslinker (Analytik Jena, Jena, Germany). In 3D cell culture, apoptosis was induced with 1 µg/mL staurosporine (S-9300, LC Laboratories, Woburn, MA, USA) and necrosis by freezing and thawing the spheroids. For ATP degradation, apyrase (A6535, Sigma-Aldrich, St. Louis, MO, USA) was used at a final concentration of 10 U/mL. ATP receptors were blocked with the P2X4 receptor inhibitor BAY-1797 (HY-130605, MedChemExpress, Princeton, NJ, USA) at 10 µM, which inhibits P2X4 and P2X7 receptors.

### 2.5. Viability Assay

LN229 cells were seeded 24 h before treatment to ensure attachment, followed by 4 h incubation with the respective compounds. Regarding PBMCs, cells were treated with RSL3 upon seeding and incubated for 48 h. After incubation, cells were stained with CellTiter Blue (G8081, Promega, Walldorf, Germany) and incubated for another hour under cell culture conditions. Afterwards, fluorescence was measured on a Tecan spark plate reader (Tecan, Männedorf, Switzerland).

### 2.6. Live-Cell Imaging (Incucyte)

Spheroids were treated and monitored for 2 days using the Incucyte^®^ S3 Live-Cell Analysis System (Sartorius, Göttingen, Germany). Images were taken every 30 min at 10× magnification. For the size increase measurement, images were analyzed using free ImageJ (Version 1.54f) Software. Size was calculated by setting the spheroid diameter in proportion to the 400 µm size bar for each picture.

### 2.7. Quantitative Real-Time PCR

For RNA analysis, three spheroids were pooled. RNA was harvested using Trizol Reagent (15596018, Invitrogen, Thermo Fisher Scientific, Waltham, MA, USA) and measured using a Nanodrop ND-1000 spectrophotometer (Peqlab, Erlangen, Germany). For reverse transcription, the Maxima First Strand cDNA Synthesis Kit for RT-PCR (Thermo Fisher Scientific) was used. Quantitative real-time PCR was performed using PowerUp SYBR Green Master Mix (A25742, Applied Biosystems, Thermo Fisher Scientific) on a QuantStudio 5 PCR Detection System (Applied Biosystems, Thermo Fisher Scientific). Relative messenger RNA expression was calculated by the ΔΔCt method and normalized to TBP. Primers are listed in Table 1.

### 2.8. Immunohistochemistry

An adaptation of the method by Pinto et al. was used [23] to prepare spheroids for embedding. Therefore, spheroids were harvested and embedded between two layers of warm Histogel (12006679, Fisher Scientific, Thermo Fisher Scientific) in Tissue-Tek cryomolds (4564, Sakura, Umkirch, Germany). Afterwards, histogel blocks were fixed in 4% paraformaldehyde solution (P087.5, Carl ROTH, Karlsruhe, Germany) for 12 h. The blocks were then dehydrated and embedded in paraffin (39602004, Leica Biosystems, Nussloch, Germany). Sections were cut with 3 µm thickness. The samples were stained with Opal Multiplex IHC Kits (Akoya Bioscience, Marlborough, MA, USA) according to manufacturer’s instructions. Primary antibodies against 4-HNE (Ab46545, Abcam, Cambridge, UK), CD4 (Ab133616, Abcam), and CD8 (M710301-2; Agilent Dako, Santa Clara, CA, USA) were used, and Opal Anti-Ms + Rb HRP (ARH1001EA, Akoya Bioscience) as secondary antibody in sequential staining. Nuclei were counterstained with DAPI (FP1490, Akoya Bioscience). Images were acquired with the Vectra3 Automated Quantitative Pathology Imaging System (Akoya Bioscience) at 20× magnification and analyzed with open QuPath 0.5.1 software [24] using positive cell detection.

### 2.9. ATP Release Assay

Extracellular ATP was measured using the RealTime-Glo™ Extracellular ATP Assay (GA5011, Promega) according to the manufacturer’s instructions. Luminescence was measured on a Tecan spark plate reader (Tecan) heated to 37 °C. Gibco RPMI media supplemented with 3% human plasma, 1% penicillin/streptomycin and buffered with 25 mM HEPES (HN77.4, Carl ROTH, Karlsruhe, Germany) was used. Samples were mixed for 1 min and measured every 10 min over a period of 24 h.

### 2.10. Spheroid Infiltration Assay

For the spheroid infiltration assay, PBMCs in RPMI containing 3% human plasma were seeded at a concentration of 0.1 × 10^6^ living cells per well and spheroid either 6 h after or simultaneously with the treatment. Spheroids were harvested after 2 days of co-culture.

### 2.11. Migration Assay

PBMCs of four different donors were pooled and 2.5 × 10^6^ living cells seeded per transwell. Sarstedt 6-well transwell inserts with 5 µm pore diameter and a pore density of 6 × 10^5^ pores/cm^2^ were used (83.3930.500, Sarstedt). In the bottom well, a tumor cell suspension was used as an attractant. Therefore, tumor cells were seeded in 15 cm cell culture dishes at a density of 2.5 × 10^6^ cells per dish one day before the experiment and incubated overnight. Tumor cells were either treated with DMSO or 1 µM RSL3 for 1.5 h followed by media replacement and a 1 h-incubation. Refer to Section 2.4 for apoptosis and necrosis induction. Afterwards, tumor cells were scraped off and the cell suspension was transferred to 6-well plates and PBMCs with or without addition of purinergic receptor inhibitor BAY-1797 (10 µM; HY-130605, MedChemExpress) were added in a transwell for 3 h at 37 °C and 5% CO_2_. Cells from inserts and wells were collected in separate tubes. To transfer all cells into test tubes the wells and inserts were washed with PBS and trypsinized for 10 min. Then, cells were stained for flow cytometric analysis.

### 2.12. Flow Cytometry and Imaging Flow Cytometry

For flow cytometric analysis, six spheroids were pooled, washed with PBS and then dissolved with accutase (A6964, Sigma-Aldrich, St. Louis, MO, USA) to receive a single-cell suspension. Samples were blocked with 2% Fc receptor binding inhibitor (130-059-901, Miltenyi Biotec, Bergisch Gladbach, Germany) and stained with Zombie dye (423108, BioLegend, San Diego, CA, USA) and fluorochrome-coupled antibodies (Table 2). The samples were measured in a FACSymphony A5SE flow cytometer (BD Bioscience, Franklin Lakes, NJ, USA) using FACSDiva software v9.6 (BD Bioscience). Data were analyzed using the FloJo^TM^ 10.10.0 Software (BD Bioscience). All primary antibodies were titrated to determine the optimal concentration. Compensation Beads (BD Bioscience) were used for single-color compensation to create multicolor compensation matrices. The gating strategy is illustrated in Supplementary Figure S1. The instrument was controlled daily by calibrations with Cytometer Setup and Tracking beads (BD Bioscience).

PD-L1 expression was analyzed by flow cytometric imaging using an Amnis ImageStreamX Mk II (Cytek, Amsterdam, The Netherlands) and analyzed with IDEAS^®^ Image Analysis Software (Version 6.3).

### 2.13. T Cell Activation Assay of Migrated PBMCs

First, a migration assay was performed. Sarstedt 6-well transwell-inserts with 5 µm pore diameter and a pore density of 6 × 10^5^ pores/ cm^2^ were used (83.3930.500, Sarstedt). 2.5 × 10^6^ living PBMCs were seeded into each transwell. As an attractant, a tumor cell suspension was used in the lower well. Therefore, tumor cells were seeded in 15 cm cell culture dishes at a density of 2.5 × 10^6^ cells per dish the day before the experiment and incubated overnight. Cells were treated with DMSO or 1 µM RSL3 6 h before they were scraped off, and the cell suspension was transferred to 6-well plates. Then, PBMCs were added to a transwell and allowed to migrate for 3 h at 37 °C and 5% CO_2_. Migrated cells were harvested, centrifuged, and the pellet was resuspended in culture medium. The cell suspension was divided into two wells, and 20 µL ImmunoCult™ Human CD3/CD28 T Cell Activator (#10971, Stemcell, Vancouver, CA, USA) were added to one well. The second one served as a control. After 2 days, the supernatants were harvested and analyzed with Cytometric Bead Array and a LEGENDplexTM Multi-Analyte Flow Assay Kit (BioLegend).

### 2.14. T Cell Activation Under Apyrase Treatment

LN229 tumor cells were seeded in 6 cm cell culture dishes at a density of 0.25 × 10^6^ cells per dish for DMSO treatment and 1 × 10^6^ cells per dish for RSL3 treatment the day before the experiment and incubated overnight. Then, cells were treated with either DMSO or 1 µM RSL3 6 h before they were scraped off, and the resulting cell suspension was transferred to 96-well plates. Subsequently, 0.1 × 10^6^ PBMCs and 10 µL ImmunoCult™ Human CD3/CD28 T Cell Activator (#10971, Stemcell) were added to each well in the presence or absence of apyrase (A6535, Sigma-Aldrich), which was used at a final concentration of 10 U/mL. After 2 days of co-culture, the supernatants were harvested and analyzed with Cytometric Bead Array and a LEGENDplex^TM^ Multi-Analyte Flow Assay Kit (BioLegend).

### 2.15. T Cell Activation with PD-L1 Knock-Down Cells

LN229 knock-down cells were seeded in 6 cm cell culture dishes one day prior to the experiment and incubated overnight. Then, cells were treated with either DMSO or 1 µM RSL3 6 h before they were scraped off. The resulting cell suspension was transferred to 96-well plates. Subsequently, 0.1 × 10^6^ PBMCs and 10 µL ImmunoCult™ Human CD3/CD28 T Cell Activator (#10971, Stemcell) were added to each well. After 2 days of co-culture, the supernatants were collected and analyzed with Cytometric Bead Array.

### 2.16. LEGENDplex^TM^ Multi-Analyte Flow Assay

Supernatants were harvested and analyzed using a customized LEGENDplex^TM^ Multi-Analyte Flow Assay Kit (BioLegend), including IL-2, IL-17A, CCL4, CCL5, CCL17, CCL18, CCL20, CCL22, TNFα, IFNγ, CXCL1, CXCL9, CXCL10, and CXCL11. The assay was performed according to the manufacturer’s instructions and measured using a FACSymphony A5SE flow cytometer (BD Bioscience) and FACSDiva v9.6 software (BD Bioscience). Data were analyzed using the LEGENDplex^TM^ Data Analysis Software (Version 2025-05-01, BioLegend).

### 2.17. Cytometric Bead Array (CBA)

BD CBA Flex Sets for Granzyme B (560304, BD Bioscience) and IL-4 (558272, BD Bioscience) and IFNγ (558269, BD Bioscience) were used. The manufacturer’s instructions (BD Cytometric Bead Array (CBA) Human Soluble Protein Master Buffer Kit Instruction Manual) were followed, except that a 0.09% sodium azide solution in PBS containing 0.5% bovine serum albumin was used for standard reconstitution and dilution of capture beads and detection reagent. Furthermore, samples were washed and resuspended with BD FACS Flow (342003, BD Bioscience) instead of wash buffer. Then, samples were measured using a FACSymphony A5SE flow cytometer (BD Bioscience) and FACSDiva v9.6 software (BD Bioscience). Data were analyzed using the FlowJo^TM^ 10.10.0 Software (BD Bioscience).

### 2.18. Western Analysis

For Western analysis, 5 spheroids were pooled and lysed in a buffer containing 4% SDS, 150 mM NaCl, and 100 mM Tris-HCl, pH 7.4, and sonicated. A protein assay kit (Bio-Rad, Munich, Germany) was used to determine protein content and 60 μg protein were loaded on a 12% SDS gel. Gels were blotted using a Trans Blot Turbo blotting system (Bio-Rad). Membranes were stained using a Revert™ 700 Total Protein Stain kit (P/N 926-11016, Licor, Lincoln, NE, USA) according to manufacturer’s advice. Afterwards, membranes were blocked and stained for PD-L1 (Ab282458, Abcam, Cambridge, UK) in 5% milk in TTBS containing 0.05% Tween 20 using a IRDye secondary antibody (LI-COR Biosciences, Bad Homburg, Germany). On an Odyssey CFx scanner (Licor) the fluorescence signal was measured and quantified with Image Studio Digits 5.0 (Licor). The signal was normalized using a lane normalization factor (LNF; intensity of a complete lane divided by the intensity of the lane with the maximal intensity). Complete pictures of total protein stains are shown in Supplementary Figures S3E and S4D.

### 2.19. Data Presentation and Statistical Analysis

Graphical data are expressed as mean ± SEM. Each biological replicate included a control and was normalized accordingly. Statistics were performed with GraphPad Prism Version 10. Statistical significance was calculated using one-way ANOVA and Tukey’s multiple comparisons test or Student’s *t*-test with * *p* ≤ 0.05, ** *p* ≤ 0.01, *** *p* ≤ 0.001, **** *p* < 0.0001. Šídák’s multiple comparisons test.

## 3. Results

### 3.1. Glioblastoma LN229 Cells Undergo Ferroptosis upon RSL3 Treatment

**Figure 1:**
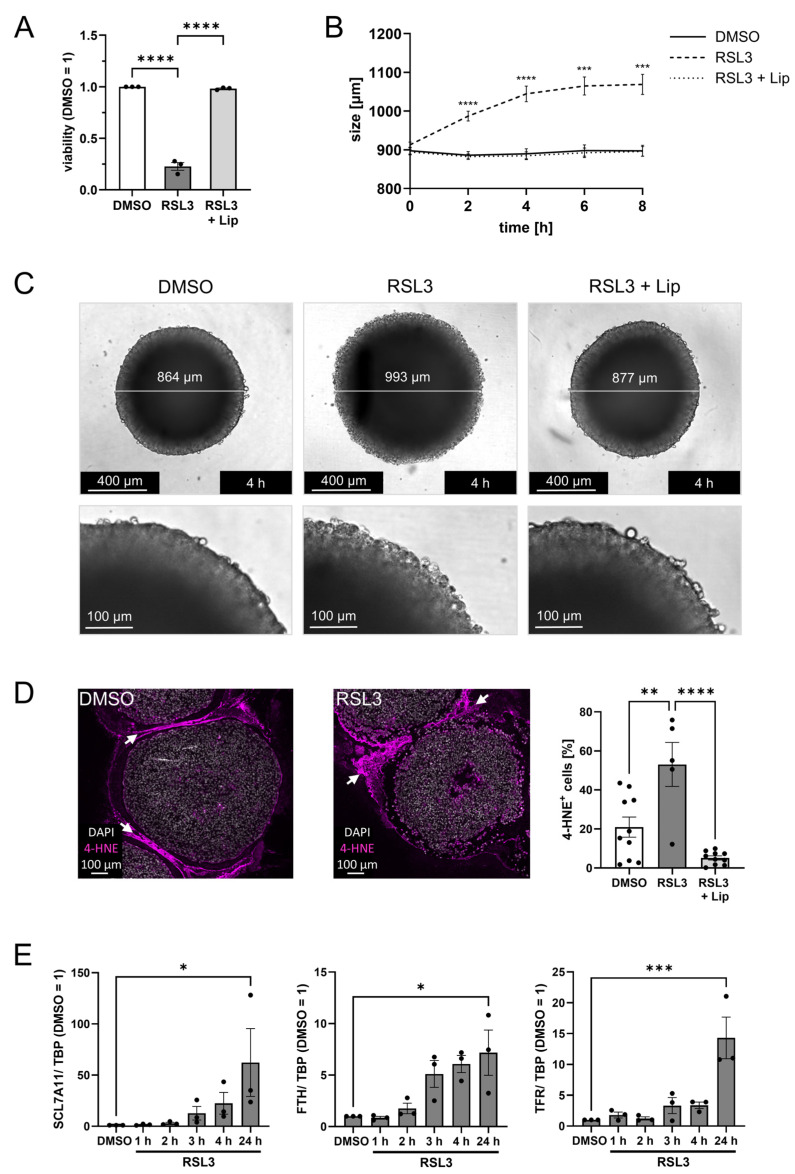
LN229 glioblastoma cells undergo ferroptosis upon RSL3 treatment. (**A**) LN229 cells were treated with 1 µM RSL3 ± 1 µM liproxstatin-1 (Lip) for 4 h, and viability was measured by CellTiter blue assay. Šídák’s multiple comparisons test was used. (**B**) LN229 spheroids were treated with RSL3 ± Lip, and spheroid size was calculated after live cell imaging for 8 h. The *p* values were calculated using 2way ANOVA and Tukey’s multiple comparisons test with * representing *p* values of the comparison of DMSO vs. RSL3. (**C**) Images of representative spheroids 4 h after stimulation with RSL3 ± Lip or DMSO as control. Scale bars indicating 400 µm. Magnifications with scale bars indicating 100 µm. (**D**) Microscopy analysis of spheroids harvested 4 h after stimulation with RSL3 or DMSO. Spheroid sections were stained with an antibody against 4-Hydroxynonenal (4-HNE). Nuclei were counterstained with DAPI. Graph shows the ratio of 4-HNE positive cells per DAPI positive cells per spheroid. Scale bars indicate 100 µm. White arrows indicate non-specific staining of the surrounding histogel. (**E**) Spheroids were treated with RSL3 for indicated times and RNA of SLC7A11, ferritin heavy chain (FTH), and transferrin receptor (TFR) was analyzed. Data were normalized to TATA box-binding protein (TBP) and DMSO control (24 h) was set to 1. For SCL7A11 Friedman test with Dunn’s multiple comparisons test was used. Data are expressed as mean values ± SEM. * *p* ≤ 0.05, ** *p* ≤ 0.01, *** *p* ≤ 0.001, **** *p* < 0.0001; *p* values were calculated using ordinary one-way ANOVA and Tukey’s multiple comparisons test if not stated otherwise.

To analyze the ability of LN229 glioblastoma cells to undergo ferroptosis, they were treated with 1 µM RSL3, which substantially reduced viability (Figure 1A). Liproxstatin-1, a lipophilic radical trapping agent and established ferroptosis inhibitor [2], completely antagonized the loss in viability, confirming a ferroptotic cell demise. To mimic 3-dimensional features of solid tumors, including hypoxic regions, a necrotic core, and nutrient gradients [25,26,27], we cultured LN229 cells as spheroids. Spheroids treated with 1 µM RSL3 were monitored for 8 h in an Incucyte device. The spheroid size significantly increased over time, reaching a plateau after 6 h (Figure 1B). Liproxstatin-1 completely abolished this effect, indicating that these changes refer to ferroptotic cell death [2]. Ferroptotic cells show morphological changes such as cytoplasmic swelling and a ballooning phenotype [1,28], which likely accounts for the increasing spheroid size during ferroptosis (Figure 1C, Supplementary Video S1). To substantiate ferroptosis in spheroids, we stained paraffin-embedded sections for 4-hydroxynonenal (4-HNE), a product of cellular lipid peroxidation. RSL3-treated spheroids showed an increased number of 4-HNE-positive cells compared to DMSO controls or a combined treatment of RSL3 with liproxstatin-1 (Figure 1D). Due to a hypoxic environment in the spheroid center, apoptosis followed by secondary necrosis forms a necrotic core [25,26,27], which likely accounts for the cell-free area, especially in RSL3-treated spheroids. RSL3-exposed spheroids were also characterized by mRNA expression changes of typical ferroptosis-associated genes such as SLC7A11, ferritin heavy chain (FTH), and transferrin receptor (TFR) [29] (Figure 1E). Their expression started to increase 2 h after RSL3 stimulation, reaching significance after 24 h. Again, this effect was sensitive to liproxstatin-1 (Supplementary Figure S2A). Conclusively, LN229 cells undergo ferroptotic cell death upon RSL3 treatment in monolayer and 3D cell culture models with a robust ferroptotic phenotype, including cell swelling, lipid peroxidation, and gene expression regulation.

### 3.2. Ferroptosis Facilitates T Cell Infiltration into Glioblastoma Spheroids

**Figure 2:**
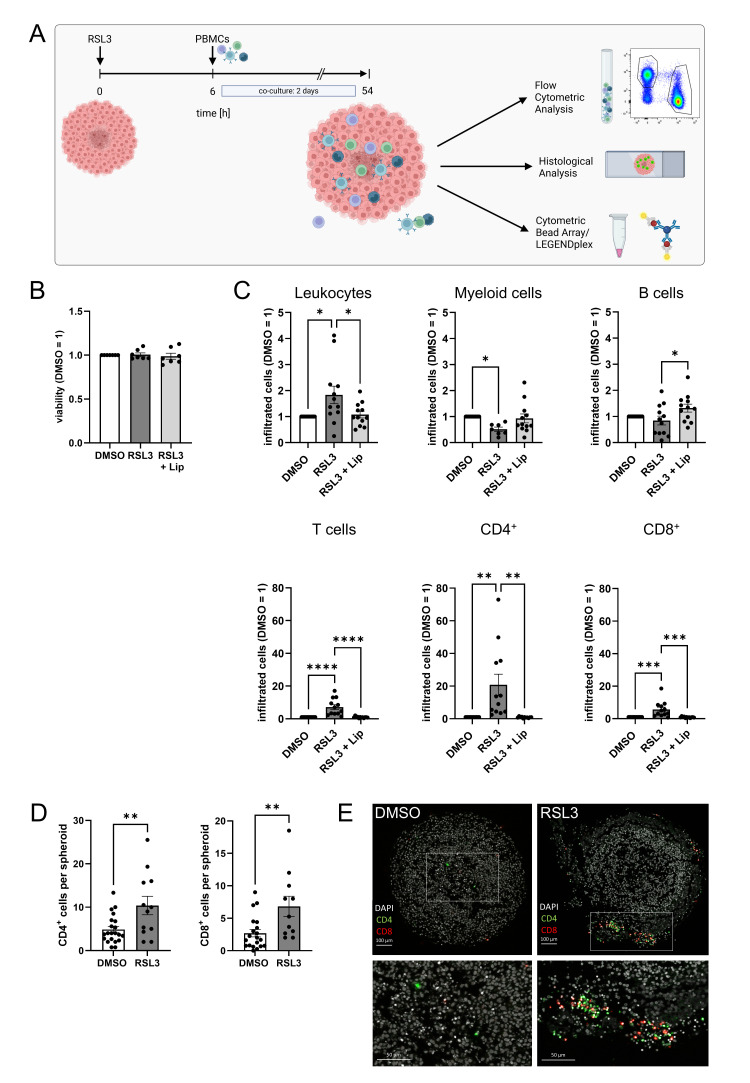
Ferroptosis enhances T cell infiltration. (**A**) Scheme of the experimental set-up for the spheroid infiltration assay. Glioblastoma cell spheroids were treated with 1 µM RSL3 and incubated for 6 h. Then, peripheral blood mononuclear cells (PBMCs) were added and co-cultured with the spheroids for a duration of 2 days. Infiltration was analyzed by flow cytometric and histological analysis. Supernatants were analyzed for immune cell activation (Figure created in BioRender. Fuhrmann, D. (2025) https://BioRender.com/3wcc3xo). (**B**) PBMCs were treated with 1 µM RSL3 ± 1 µM liproxstatin-1 (Lip) for 48 h and viability was assessed by CellTiter blue assay. Data from 7 individual donors. (**C**) PBMC populations infiltrating into living and ferroptotic spheroids were analyzed by flow cytometry after 2 days of co-culture. Cell counts were normalized to the DMSO-treated control, which was set to 1. Data from 12 individual donors. (**D**) Ferroptotic and living spheroids infiltrated by PBMCs were stained for CD4 and CD8, and the number of positive cells per spheroid was counted. Unpaired *t* test *p* values. (**E**) Microscopy images of PBMC-infiltrated ferroptotic and living spheroids stained for CD4 and CD8 after 2 days of co-culture. Nuclei were counterstained with DAPI. Scale bars indicate 100 µm. Magnifications with scale bars indicating 50 µm. Data are expressed as mean values ± SEM. * *p* ≤ 0.05, ** *p* ≤ 0.01, *** *p* ≤ 0.001, **** *p* < 0.0001; *p* values were calculated using ordinary one-way ANOVA and Tukey’s multiple comparisons test if not stated otherwise.

To explore how ferroptosis in LN229 spheroids affects immune cell infiltration, we isolated primary human peripheral blood mononuclear cells (PBMCs) from buffy coats and added them to RSL3-treated spheroids. After 2 days of co-culture, infiltrated cells were analyzed by flow cytometry and histology. The experimental setup is visualized in Figure 2A. Control experiments revealed that PBMCs, treated with 1 µM RSL3 for 2 days, did not lose cell viability (Figure 2B). To determine immune cell populations attracted by ferroptotic tumor cells, we treated glioblastoma spheroids with RSL3 and added PBMCs 6 h after stimulation, the time point when ferroptosis was fully established. Infiltration of leukocytes into ferroptotic spheroids increased compared to a DMSO control or RSL3/liproxstatin-1 treatment (Figure 2C). Specifically, T cell infiltration into ferroptotic spheroids increased 7-fold compared to controls. This effect occurred with CD8^+^ and CD4^+^ T cells but was more prominent with CD4^+^ T cells, with a 20-fold increased infiltration of CD4^+^ T cells compared to a 5-fold effect seen for CD8^+^ T cells. Infiltration of myeloid cells and B cells was slightly reduced upon ferroptosis of LN229 spheroids. Histology of spheroids two days after PBMC infiltration corroborated our flow cytometry data (Figure 2D,E). The number of CD4^+^ and CD8^+^ cells in ferroptotic spheroids was higher compared to DMSO controls. T cells were mainly localized in the outer layer of spheroids.

### 3.3. Ferroptotic Cells Attract T Cells More Efficiently than Other Forms of Cell Death

**Figure 3:**
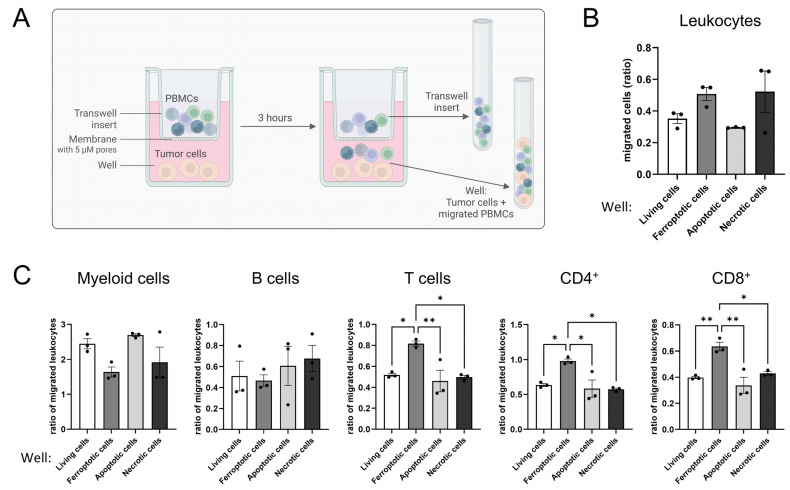
Ferroptotic but not apoptotic or necrotic cells attract T cells. (**A**) Scheme of the experimental setup for the migration assay. Peripheral blood mononuclear cells (PBMCs) were allowed to migrate the insert membrane towards living, ferroptotic (1 µM RSL3), apoptotic (UV irradiation), or necrotic (freeze and thaw) tumor cells for 3 h. Afterwards migrated cells in the well and remaining cells in the transwell were harvested and analyzed separately by flow cytometry (Figure Created in BioRender. Fuhrmann, D. (2025) https://BioRender.com/9pvj2s6). (**B**) Leukocytes migrating towards living, ferroptotic, apoptotic, and necrotic tumor cells were analyzed by flow cytometry. (**C**) PBMC populations migrating towards living, ferroptotic, apoptotic, and necrotic tumor cells were analyzed by flow cytometry. PBMC populations were normalized to the respective number of migrated leukocytes in each individual sample to generate comparability among the different treatments. Holm-Šídák’s multiple comparisons test was used. Data are expressed as mean values ± SEM. * *p* ≤ 0.05, ** *p* ≤ 0.01; *p* values were calculated using ordinary one-way ANOVA and Tukey’s multiple comparisons test if not stated otherwise.

Tumor cell spheroids are a 3D in vitro model with the advantage of mimicking tumor apoptosis and necrosis due to a hypoxic core area [25,26,27]. To analyze the distinct roles of ferroptosis, apoptosis, and necrosis for T cell migration, we performed transwell migration assays. Living or dying glioblastoma cells were cultured in 6-well plates with PBMCs added to the transwell inserts above tumor cells, allowing them to migrate for 3 h. For analysis, migrated cells and cells remaining in the inserts were determined by flow cytometry (Figure 3A). An elevated migration of leukocytes towards ferroptotic and necrotic cells compared to apoptotic or living cells was observed (Figure 3B). Next, we focused on the analysis of individual leukocyte subsets. To exclude a donor-dependent variability in leukocyte numbers, we normalized the number of migrated leukocyte subsets to the number of migrated leukocytes. T cells, including CD4^+^ and CD8^+^ subsets, migrated more effectively towards ferroptotic cells compared to apoptotic, necrotic, and living cells, while myeloid and B cells showed minor or no differences comparing ferroptotic vs. living cells. Rather, myeloid and B cells migrated better towards apoptotic than ferroptotic cells (Figure 3C).

Apparently, ferroptotic but neither apoptotic nor necrotic tumor cells attracted T cells. As these effects become apparent in a transwell migration setup, they argue for the involvement of a soluble factor, possibly a DAMP.

### 3.4. Death by Ferroptosis Releases More ATP than Apoptosis

**Figure 4:**
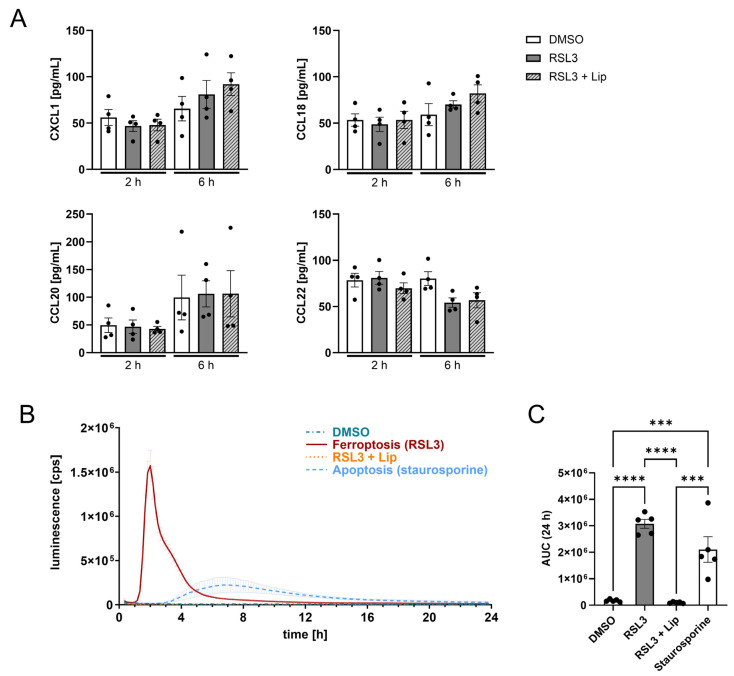
Ferroptosis releases more ATP from dying cells than apoptosis. (**A**) LN229 spheroids were treated with 1 µM RSL3 ± 1 µM liproxstatin-1 (Lip) for 2 and 6 h. Supernatants were analyzed by multiplex immunoassay for CXCL1, CCL18, CCL20 and CCL22. (**B**) ATP release over 24 h from LN229 glioblastoma spheroids treated with RSL3 ± liproxstatin-1 or staurosporine (1 µg/mL). (**C**) Quantification of the total ATP release using the area under the curve (AUC) from B. Data are expressed as mean values ± SEM. *** *p* ≤ 0.001, **** *p* < 0.0001; *p* values were calculated using ordinary one-way ANOVA and Tukey’s multiple comparisons test if not stated otherwise.

Exploring how ferroptotic glioblastoma cells attract T cells, we measured chemokines, cytokines, and ATP in the supernatants of glioblastoma spheroids harvested 2 and 6 h after RSL3-treatment. While most of the analyzed chemokines (listed in Section 2) were not released by ferroptotic glioblastoma cells, we detected variations for CXCL1, CCL18, CCL20, and CCL22 (Figure 4A); however, these were not significant when comparing RSL3 to RSL3/liproxstatin-1.

To understand T cell recruitment, extracellular ATP (eATP) levels were followed over 24 h after ferroptosis induction. eATP is a potent DAMP, released by stressed or dying cells [16]. By investigating ATP release from dying cells in spheroids, we sought to emphasize the divergence between ferroptosis and apoptosis. Ferroptotic spheroids profoundly released ATP with a peak 2 h after RSL3-treatment, which was roughly 9-fold higher compared to apoptosis. The maximum of ATP release from apoptotic cells occurred at around 7.5 h, with prolonged release of ATP over 12 h. ATP release from ferroptotic cells only lasted around 6 h (Figure 4B). The quantity of ATP being released from ferroptotic cells over 24 h was 1.7-fold higher compared to apoptotic cells, while living cells or RSL3/liproxstatin-1 cotreatment provoked no ATP release (Figure 4C). Possibly, the ATP release from ferroptotic cells accounted for T cell recruitment.

### 3.5. ATP Causes T Cell Infiltration into Glioblastoma Spheroids

**Figure 5:**
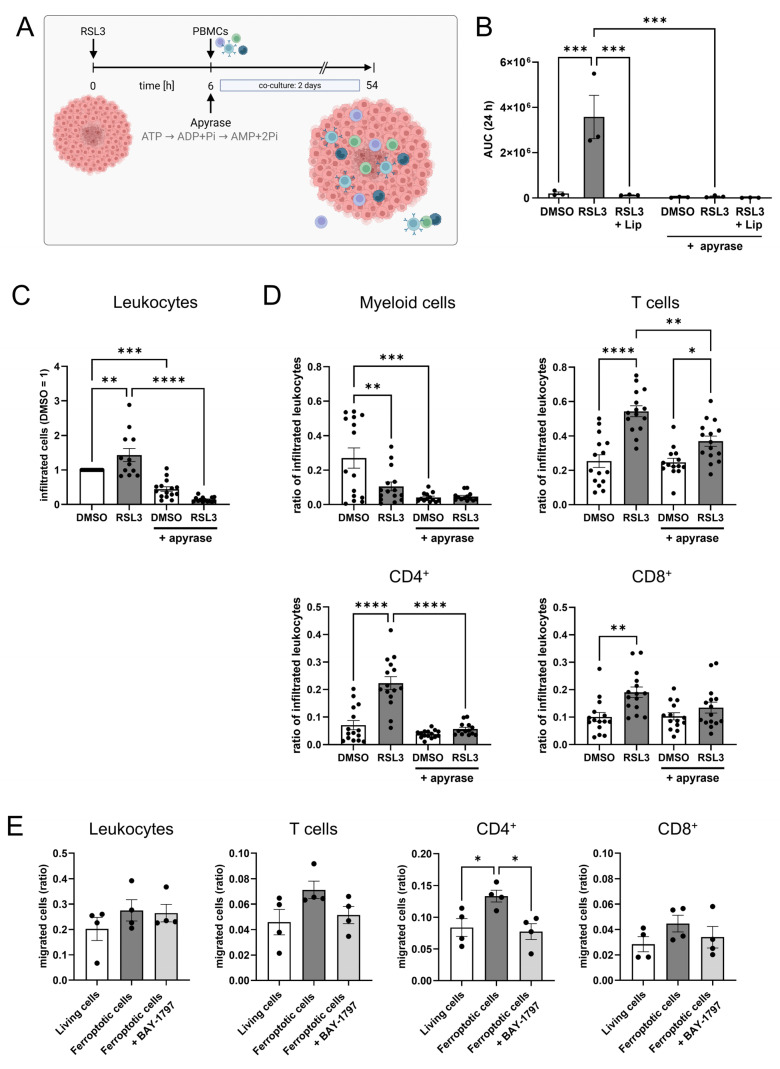
ATP mediates T cell infiltration into glioblastoma spheroids. (**A**) Schematic outline of the experimental setup for the spheroid infiltration assay including ATP degradation by apyrase (Figure created in BioRender. Fuhrmann, D. (2025) https://BioRender.com/8nr6inp). (**B**) LN229 glioblastoma spheroids were treated with 1 µM RSL3 ± 1 µM liproxstatin-1 (Lip) and apyrase (10 U/mL). ATP release was measured over 24 h and visualized as area under the curve (AUC). (**C**) Leukocytes infiltrating DMSO- and RSL3-treated spheroids, with and without addition of apyrase, were analyzed by flow cytometry after 2 days of co-culture. Data from 15 individual donors. (**D**) Populations of peripheral blood mononuclear cells (PBMCs) infiltrating DMSO- and RSL3-treated spheroids, with and without addition of apyrase, were analyzed by flow cytometry after 2 days of co-culture. PBMC populations were normalized to the respective number of infiltrated leukocytes in each individual sample to generate comparability among the different treatments. Data from 15 individual donors. (**E**) PBMCs were incubated with BAY-1797 (10 µM), a purinergic receptor inhibitor, prior to migration towards living and ferroptotic (1 µM RSL3) glioblastoma cells. Cell migration was analyzed by flow cytometry after 3 h. Data are expressed as mean values ± SEM. * *p* ≤ 0.05, ** *p* ≤ 0.01, *** *p* ≤ 0.001, **** *p* < 0.0001; *p* values were calculated using ordinary one-way ANOVA and Tukey’s multiple comparisons test if not stated otherwise.

To investigate the effect of eATP on T cell motility, we performed spheroid infiltration assays upon eATP depletion by apyrase, a highly active ATP-diphosphohydrolase that catalyzes the sequential hydrolysis of ATP to ADP and AMP (Figure 5A). First, we confirmed ATP depletion by apyrase. Apyrase completely abolished ATP detection in the supernatant of RSL3-treated spheroids (Figure 5B). We then added apyrase to glioblastoma spheroids co-cultured with PBMCs. Leukocyte infiltration significantly increased upon RSL3-treatment as seen before but decreased significantly when apyrase was added (Figure 5C). Interestingly, apyrase itself reduced leukocyte infiltration in DMSO controls, implying a role of ATP for leukocyte migration also under basal conditions. To compare infiltration of leukocyte subsets, we normalized the number of infiltrated leukocyte subsets to the total amount of infiltrated leukocytes. Apyrase lowered infiltration of myeloid cells into DMSO-treated spheroids, which was expected as eATP is a known chemoattractant for monocytes [30,31]. For T cell infiltration, the increase upon RSL3 treatment was recapitulated. This was significantly reduced in apyrase and RSL3-treated co-cultures (Figure 5D), while infiltration in DMSO controls remained unaffected. Regarding T cell subset infiltration, CD4^+^ cells were significantly affected by apyrase treatment. Also, CD8^+^ cells revealed a significantly increased infiltration upon RSL3 treatment, which was lowered by apyrase, without reaching significance. Having verified the role of ATP for T cell attraction by ferroptotic cells, we pharmacologically blocked purinergic receptors (P2X4, P2X7 and P2X3) on leukocytes with BAY-1797 and determined migration towards ferroptotic cells in a transwell assay. Here, leukocytes showed a slight increase in migration towards ferroptotic cells, but there was no difference with and without the inhibitor (Figure 5E). T lymphocytes showed an increased migration towards ferroptotic cells, which was antagonized by BAY-1797. This effect was recapitulated by CD4^+^ and CD8^+^ T cells, but only the CD4^+^ subset reached significance.

To conclude, RSL3 induced ferroptosis in glioblastoma spheroids with a robust ferroptotic phenotype, that attracted T cells. This effect was mediated by the release of ATP and pronounced in CD4^+^ cells.

### 3.6. T Cell Activation Is Suppressed by Ferroptotic Cells

**Figure 6:**
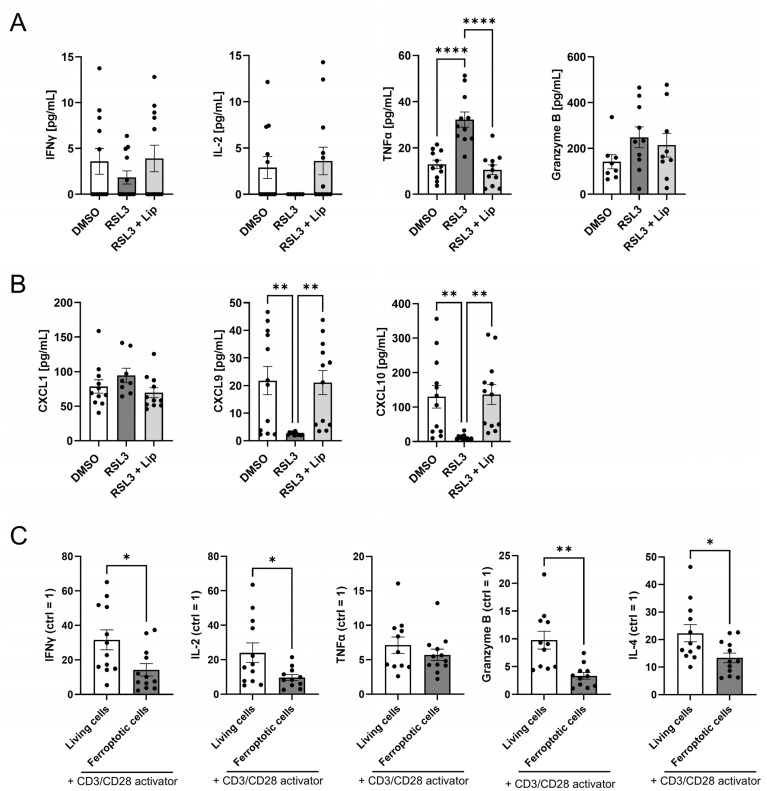
Ferroptotic cells suppress T cell activation. (**A**) Interferon gamma (IFNγ), interleukin 2 (IL-2), tumor necrosis factor α (TNFα), and granzyme B were analyzed by multiplex immunoassays in supernatants of peripheral blood mononuclear cells (PBMCs) and RSL3-treated spheroids after 2 days of co-culture. Data from 12 individual donors. (**B**) Supernatants of PBMCs and RSL3-treated spheroids after 2 days of co-culture. CXCL1, CXCL9, and CXCL10 were analyzed by multiplex immunoassays in supernatants of PBMCs co-cultured with RSL3-treated spheroids after 2 days. Data from 12 individual donors. (**C**) Stimulation of T cells with a CD3/CD28 activator after migration of PBMCs towards living or ferroptotic (1 µM RSL3) glioblastoma cells for 2 days. Interferon gamma (IFNγ), interleukin 2 (IL-2), interleukin 4 (IL-4), tumor necrosis factor α (TNFα), and granzyme B in supernatants were analyzed by multiplex immunoassays in supernatants. The respective control containing no activator for each individual sample and treatment was set to 1. Data from 12 individual donors, unpaired t test *p* values. Data are expressed as mean values ± SEM. * *p* ≤ 0.05, ** *p* ≤ 0.01, **** *p* < 0.0001; *p* values were calculated using ordinary one-way ANOVA and Tukey’s multiple comparisons test if not stated otherwise.

T cell activation upon antigen recognition and subsequent cytokine release is crucial for an efficient anti-tumor response [22,32,33]. Since ferroptosis increased the number of T lymphocytes in our glioblastoma spheroid model, we explored their state of activation. T cell activation was assessed in supernatants of PBMCs and glioblastoma spheroid co-cultures. Regarding CD4^+^ T cell activation, we measured IFNγ, IL-2 and TNFα levels. Of these cytokines, only TNFα increased in co-cultures with ferroptotic spheroids, but levels were generally low. For CD8^+^ T cell activation, we measured Granzyme B release, which was not significantly altered in RSL3-treated co-culture and could not be antagonized by liproxstatin-1 (Figure 6A). Furthermore, we checked inflammation-related chemokines. CXCL1 secretion did not differ between treatments. The release of CXCL9 and CXCL10 was significantly lower in co-cultures with RSL3-treated spheroids (Figure 6B). We also examined whether the contact with ferroptotic tumor cells altered T cell activation after migration. Experimentally, PBMCs were allowed to migrate either to ferroptotic or living glioblastoma cells. Migrated cells were then cultured with or without a CD3/CD28 activator for 2 days, and supernatants were subsequently analyzed for secreted T cell activation markers by multiplex immunoassay. Except for TNFα, which showed no effect upon treatment with CD3/CD28, levels of INFγ, IL-2, IL-4, and granzyme B increased upon CD3/CD28 activation in the DMSO-treated co-cultures. This increase was significantly lower in RSL3-treated samples. (Figure 6C). Conclusively, ferroptotic glioblastoma cells prevent T cell activation.

### 3.7. Apyrase Treatment Facilitates T Cell Responses and ATP Partially Inhibits T Cell Activation

**Figure 7:**
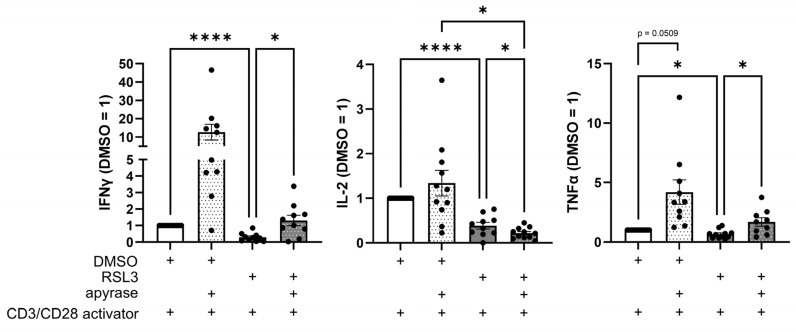
Apyrase treatment facilitates T cell response. CD3/CD28-activated peripheral blood mononuclear cells (PBMCs) and RSL3-treated LN229 glioblastoma cells were co-cultured in the presence or absence of apyrase (10 U/mL) for 2 days. Levels of Interferon gamma (IFNγ), interleukin 2 (IL-2), and tumor necrosis factor α (TNFα) in supernatants were analyzed by multiplex immunoassay and quantified by normalizing to DMSO controls. Data from 11 individual donors. Data are expressed as mean values ± SEM. * *p* ≤ 0.05, **** *p* < 0.0001; *p* values were calculated using ordinary one-way ANOVA and Tukey’s multiple comparisons test if not stated otherwise.

Next, we reached out to understand how ferroptotic cells might impede T cell activation. Since eATP depletion affected T cell infiltration, we sought to investigate its influence on T cell activation. PBMCs and living or ferroptotic glioblastoma cells were co-cultured with or without apyrase in the presence of a CD3/CD28 activator. After 2 days, supernatants were analyzed for secreted T cell activation markers by multiplex immunoassay (Figure 7). Interestingly, levels of IFNγ and TNFα increased upon apyrase addition, whereas IL-2 levels further decreased in the RSL3- and apyrase-treated co-culture. This data suggests an inhibitory function of eATP on IFNγ-secreting CD4^+^ T cells, but shows that eATP is not the single opponent of T cell activation produced by ferroptotic cells.

Taken together, RSL3 induced ferroptosis in glioblastoma spheroids with a robust ferroptotic phenotype that attracted T cells through ATP released from ferroptotic cells. Ferroptotic cells inhibited T cell activation partially through extracellular ATP.

## 4. Discussion

Glioblastoma remains one of the most treatment-resistant cancers, in part due to its immunosuppressive microenvironment and poor T cell infiltration [21,22,34]. This scenario accounts for poor immunotherapy responses [34]. As an emerging form of distinct regulated cell death, ferroptosis has gained attention for its potential to overcome therapeutic resistance [1,35]. Elevated levels of reactive oxygen species levels are a common feature of cancer cells and glioblastoma cells in particular [36]. Thus, blocking the antioxidant defense by ferroptosis inducers unlashes substantial oxidative stress, which leads to cell death. Nevertheless, directing drugs to distinct target tissues is a problem which needs to be solved. To further enhance treatment selectivity, recent studies have explored nanoconjugates [37] and other nanoparticle-based delivery systems for the targeted delivery of molecules or microRNAs to drive ferroptosis in tumor cells [38]. In this study, we investigated how ferroptosis in glioblastoma spheroids influences immune cell infiltration and activation.

Solid tumors are dense structures characterized by a complex microenvironment. To understand how ferroptosis operates within tissue-like structures, 3D models, such as spheroids, seem advantageous. We first confirmed that glioblastoma spheroids reliably undergo ferroptosis upon RSL3 treatment, despite the resistance typically associated with high cell density [39,40,41]. Lipid peroxidation, a signature of ferroptosis, occurred both at the spheroid periphery and within inner layers, suggesting a propagation of ferroptosis, which was observed in murine kidney tubules [42], where ferroptotic death spreads between adjacent cells via lipid peroxidation across cell membranes [41,43].

Migration of immune cells towards DAMPs released by dead cells is a crucial process for mounting an immune response, resolving inflammation and facilitating tissue regeneration [10,30]. Ferroptotic glioblastoma cells significantly increased T cell infiltration, with a pronounced effect on CD4^+^ T cells. This observation was reproducible in a transwell assay, indicating the involvement of a soluble chemotactic factor. While known T cell attractants were not specifically elevated, we identified ATP as a candidate DAMP released during ferroptosis, as ferroptotic cells release high amounts of ATP and display distinct kinetics compared to apoptotic cells. This effect may be explained by the fact that DAMP release is highly dependent on the type, phase, and trigger of cell death [11,44,45]. Moreover, the release mechanism and spatiotemporal dynamics of the same signaling molecule can vary, potentially altering its chemotactic properties and immune-modulating effects [11].

ATP is primarily an intracellular energy carrier, but upon tissue damage or stress, its release into the extracellular space allows it to function as a DAMP and immune signaling molecule [10,12,46]. Extracellular ATP is known to influence immune cell migration and activation via purinergic signaling [47,48,49]. We found that CD4^+^ T cells were more responsive to eATP than CD8^+^ T cells, and both ATP depletion by apyrase and purinergic receptor inhibition by BAY-1797 reduced their migration. These results are in line with outcomes of previous studies, for example, by Aswad et al. which showed a higher sensitivity of murine CD4^+^ T cells to eATP than CD8^+^ cells, due to different receptor expression patterns and different sensitivity to receptor stimulation [50,51]. Despite enhanced infiltration and DAMP release, T cell activation remained impaired in co-cultures with ferroptotic cells.

T cell infiltration and activation are crucial processes in the immune defense against cancer, as high T cell numbers often correlate with better immunotherapy responses [52,53]. However, these effects depend on the infiltrated T cell’s type and functional state, with activation failure often resulting from T cell exhaustion driven by immunosuppressive cells like regulatory T cells [22,53,54,55,56]. In our system, impaired T cell activation was not due to regulatory T cells, whose numbers declined in ferroptotic spheroids (Supplementary Figure S2B), possibly because of their heightened sensitivity to ATP-induced cell death, as was shown in mice [50]. Furthermore, T cell activation can be diminished by dysregulated purinergic signaling, e.g., by eATP and expression of inhibitory proteins [53]. The role of eATP and its receptors in T cell activation is discussed controversially in the literature and seems to be strongly tissue- and context-dependent, ranging from promoting migration and activation to cell death induction [47,48,49,50,57]. While depletion of eATP partially restored CD4^+^ T cell activation, it did not fully rescue cytokine production in our system, indicating that eATP reduces T cell activation but additional immunosuppressive mechanisms must be at play.

PD-L1, a key immune checkpoint molecule, is known to be upregulated during ferroptosis [35,58,59,60]. Ferroptotic glioblastoma cells increased PD-L1 expression, but inhibition and knockdown of PD-L1 failed to restore T cell activation (Supplementary Figures S3 and S4). These findings suggest that PD-L1 does not account for the impairment of T lymphocyte activation under ferroptotic conditions and means that attraction does not necessarily go in line with activation. Consequently, tumor therapy by induction of ferroptosis may need additional treatments to facilitate immune cell activation. Further research on the interplay between ferroptosis and immune cells is essential, particularly in the context of glioblastoma treatment, as current understanding how ferroptotic cell death influences immune cell behavior within the tumor microenvironment remains still limited. As the current findings are based on in vitro experiments, future studies are needed to confirm these observations and to establish their clinical significance. Further, suppressive factors besides ATP release by ferroptotic cells appear likely and need further attention. Given the highly immunosuppressive nature of glioblastoma and its resistance to conventional immunotherapies, uncovering how ferroptosis impairs immune cell activation and function could reveal novel therapeutic strategies to enhance anti-tumor immunity and overcome treatment resistance.

## 5. Conclusions

We conceptualized a study to explore the impact of ferroptosis on immune cell migration and activation in a glioblastoma spheroid tumor model. While ferroptotic glioblastoma cells attracted CD4^+^ T lymphocytes by releasing ATP, their activation was inhibited because of extracellular ATP.

## Figures and Tables

**Table 1:** List of primers.

Target	Accession	Forward	Reverse
TBP	NM_003194.5	GCATCACTGTTTCTTGGCGT	CGCTGGAACTCGTCTCACTA
TFR	NM_003234.4	GAGCGTCGGGATATCGGGT	CAGGATGAAGGGAGGACACG
FTH	NM_002032.3	TGACAAAAATGACCCCCATT	CAGGGTGTGCTTGTCAAAGA
SLC7A11	NM_014331.4	GGTCCATTACCAGCTTTTGTACG	AATGTAGCGTCCAAATGCCAG
PD-L1	NM_014143.4/.2	TGGCATTTGCTGAACGCATTT	TGCAGCCAGGTCTAATTGTTTT

**Table 2:** Antibody list.

Target	Article Description	Art. No.	Supplier
CD4	BD HorizonTM Customs BB630-P2 Mouse anti-human CD4	Custom-made	BD Bioscience
CD3	BD HorizonTM BUV805 Mouse anti-human CD3	612893	BD Bioscience
CD8	BD HorizonTM BV650 Mouse anti-human CD8	563821	BD Bioscience
CD20	BD PharmingenTM APC-H7 Mouse anti-human CD20	560853	BD Bioscience
CD25	BD PharmingenTM PE-CyTM7 Mouse Anti-human CD25	557741	BD Bioscience
CD33	BD HorizonTM BV510 Mouse anti-human CD33	563257	BD Bioscience
CD45	BD OptiBuildTM BUV661 Mouse anti-human CD45	750178	BD Bioscience
CD56	Brilliant Violet 605TM anti-human CD56 (NCAM)	318334	BioLegend
CD123	PE/Cyanine5 anti-human CD123	306008	BioLegend
CD127	BD HorizonTM BB700 Mouse anti-human CD127	566398	BD Bioscience
CD274	APC anti-human CD274 (B7-H1, PD-L1)	329707	BioLegend
HLA-DR	APC/FireTM 750 anti-human HLA-DR	307658	BioLegend

## Data Availability

The original contributions presented in this study are included in the article/Supplementary Material. Further inquiries can be directed to the corresponding author.

## Supplementary Materials

The following supporting information can be downloaded at: https://www.mdpi.com/article/10.3390/antiox14111373/s1, Video S1: Ferroptosis in glioblastoma spheroids; Figure S1: Gating strategy for flow cytometric analysis; Figure S2: Liproxstatin rescue; Figure S3: PD-L1 expression increases during ferroptosis; Figure S4: Ferroptotic cells do not impede T cell activation via PD-L1.

## Abbreviations

The following abbreviations are used in this manuscript:

4-HNE	4-hydroxynonenal
ADP	Adenosine diphosphate
AMP	Adenosine monophosphate
ATP	Adenosine triphosphate
AUC	Area under the curve
CBA	Cytometric bead array
CCL	C-C motif chemokine ligand
CD	Cluster of differentiation
cDNA	Complementary deoxyribonucleic acid
CXCL	C-X-C motif chemokine ligand
DAMPs	Danger-associated molecular patterns
DAPI	4′,6-Diamidino-2-phenylindole
DMSO	Dimethyl sulfoxide
eATP	Extracellular adenosine triphosphate
EDTA	Ethylenediaminetetraacetic acid
FTH	ferritin heavy chain
HEPES	N-2-Hydroxyethylpiperazine-N’-2-ethane sulphonic acid
HLA-DR	Human leucocyte antigen-DR
IFN	Interferon
IL	interleukin
Lip	Liproxstatin-1
PBMCs	Peripheral blood mononuclear cells
PBS	Phosphate-buffered saline
PD-L1	Programmed death-ligand 1
(m)RNA	(messenger) Ribonucleic acid
RPMI	Roswell Park Memorial Institute
RSL3	Ras-selective lethal small molecule 3
RT-PCR	Reverse transcription polymerase chain reaction
SLC7A11	Solute carrier family 7 member 11
TBP	TATA box-binding protein
TFR	transferrin receptor
TNF	Tumor necrosis factor
