# Supplementary material for: Ferroptosis Enhances T Lymphocyte Infiltration into Glioblastoma Spheroids
Source: Antioxidants (Basel). 2025 Nov 19;14(11):1373. doi: 10.3390/antiox14111373 (PMC12649233; doi:10.3390/antiox14111373)
Supplement: Supplementary file 1 [file antioxidants-14-01373-s001.zip › Supplementary Data.pdf]

Supplementary Materials:

Video S1: Ferroptosis in glioblastoma spheroids.

LN229 glioblastoma spheroids were incubated with 1 μM RSL3, either alone or in combination with 1 μM liproxstatin-1 as a negative control. Images were captured at hourly intervals for 12 hours using the Incucyte live-cell imaging device to monitor morphological changes over time. In each image, a size scale is shown on the left (whole size bar equals 400 μm), and the time of image acquisition is indicated on the right.

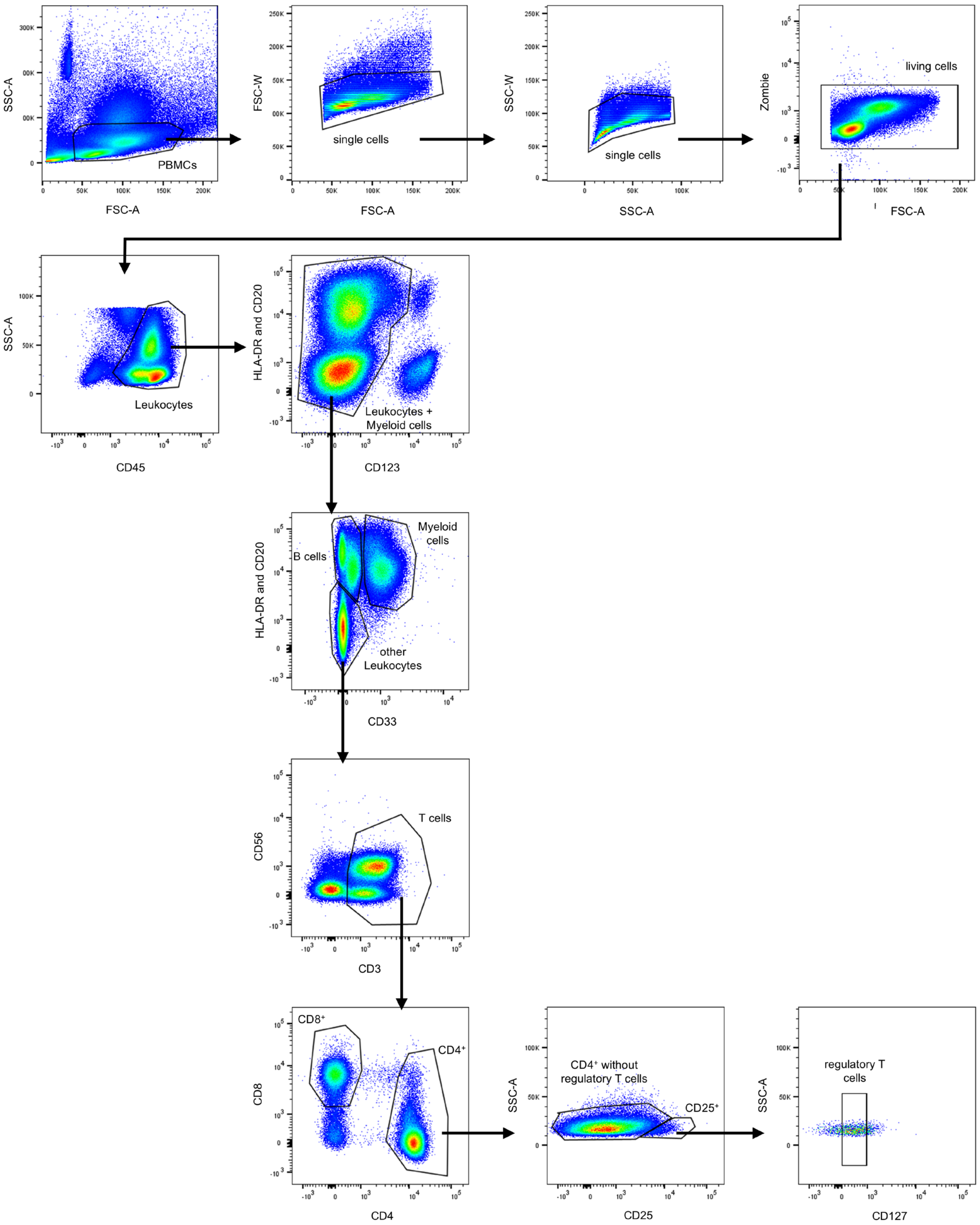

Figure S1. Gating strategy for flow cytometric analysis.

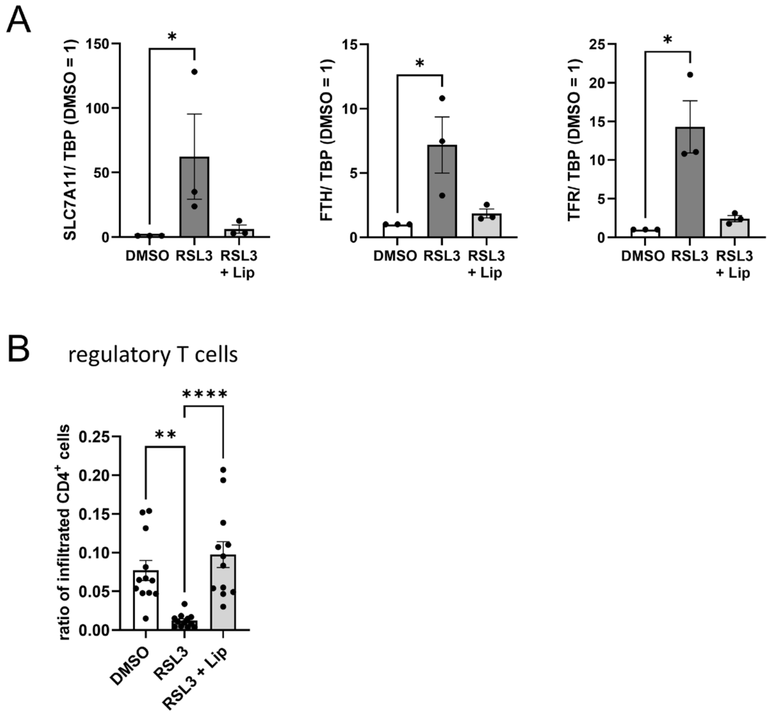

Figure S2. A Spheroids were treated with RSL3 for 24 hours and RNA of SLC7A11, ferritin heavy chain (FTH), and transferrin receptor (TFR) was analyzed. Data were normalized to TATA box binding protein (TBP) and DMSO control was set to 1. Dunn's multiple comparisons test was used. **B** Regulatory T cells (here defined as $CD4^+CD25^+CD127^{low}$) infiltrating into living and ferroptotic spheroids were analyzed by flow cytometry after 2 days of co-culture. Data normalized to infiltrated $CD4^+$ cells of each sample from 12 individual donors.

Data are expressed as mean values ± SEM. *$p \le 0.05$, **$p \le 0.01$, ***$p \le 0.001$, ****$p < 0.0001$; p values were calculated using ordinary one-way ANOVA and Tukey's multiple comparisons test if not stated otherwise.

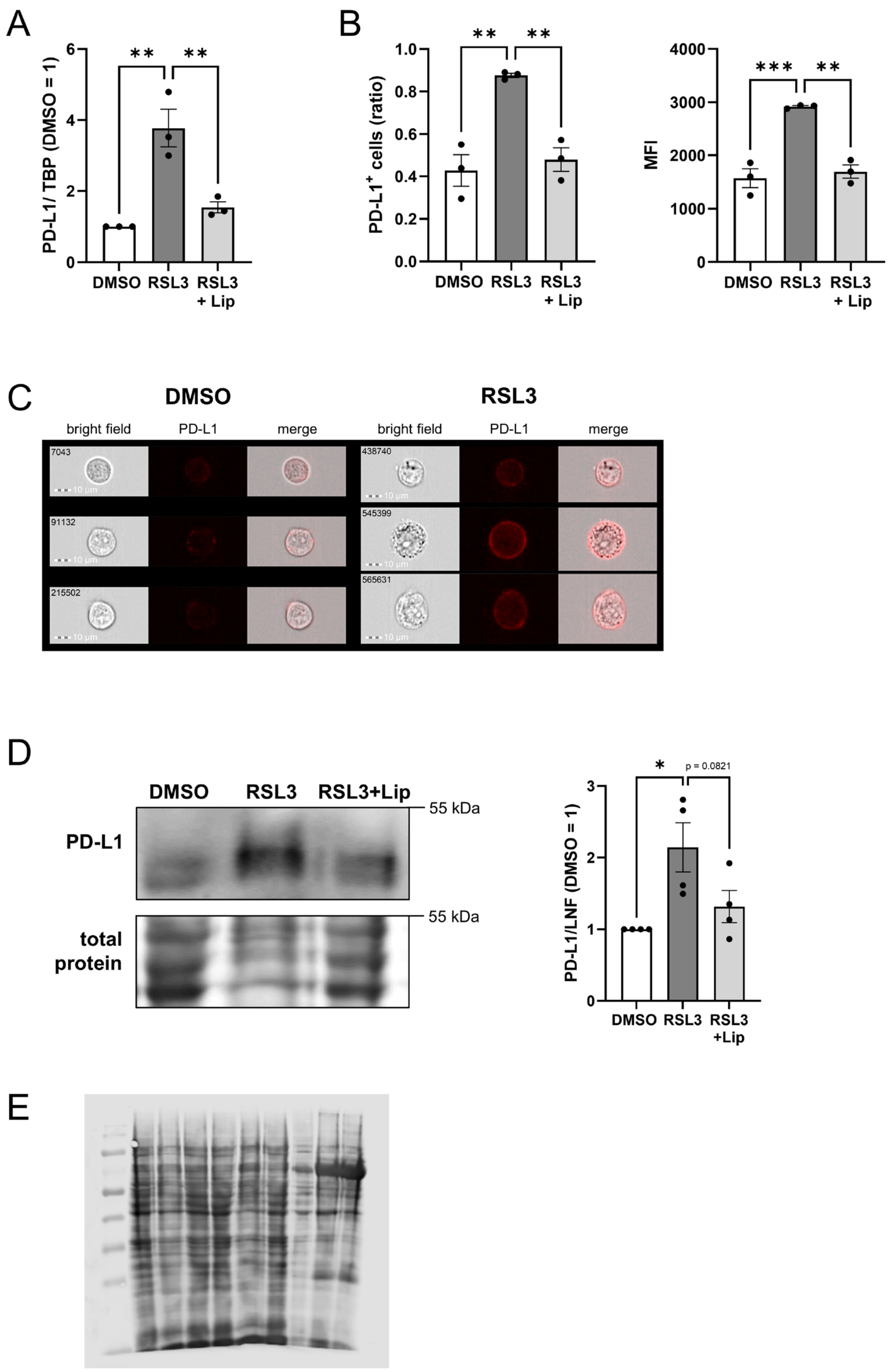

Figure S 3. PD-L1 expression increases during ferroptosis.

LN229 spheroids were treated with 1 µM RSL3 ± 1 µM liproxstatin-1 (Lip) for 24 hours.

A RNA of programmed cell death ligand-1 (PD-L1) was analyzed and normalized to TATA box binding protein (TBP). DMSO control was set to 1. **B** Spheroids were stained for PD-L1 and analyzed by flow cytometry. PD-L1 positive cells were counted and median fluorescence intensity (MFI) was compared between living and ferroptotic spheroids. **C** Spheroids were stained for PD-L1 and analyzed by imaging flow cytometry. Images of three representative cells are shown. Scale bars indicate 10 µm. Numbers included in the picture indicate the event count of the flow cytometer. **D** Spheroid lysates were analyzed for PD-L1 by Western analyses. Data were normalized to the lane normalization factor. **E** Complete total protein stain for Western blot shown in Fig. S3 D. A Revert total protein

stain was used, and membranes were scanned on an Odyssey CFx scanner.

Data are expressed as mean values ± SEM. *p ≤ 0.05, **p ≤ 0.01, ***p ≤ 0.001, ****p < 0.0001; p values were calculated using ordinary one-way ANOVA and Tukey's multiple comparisons test if not stated otherwise.

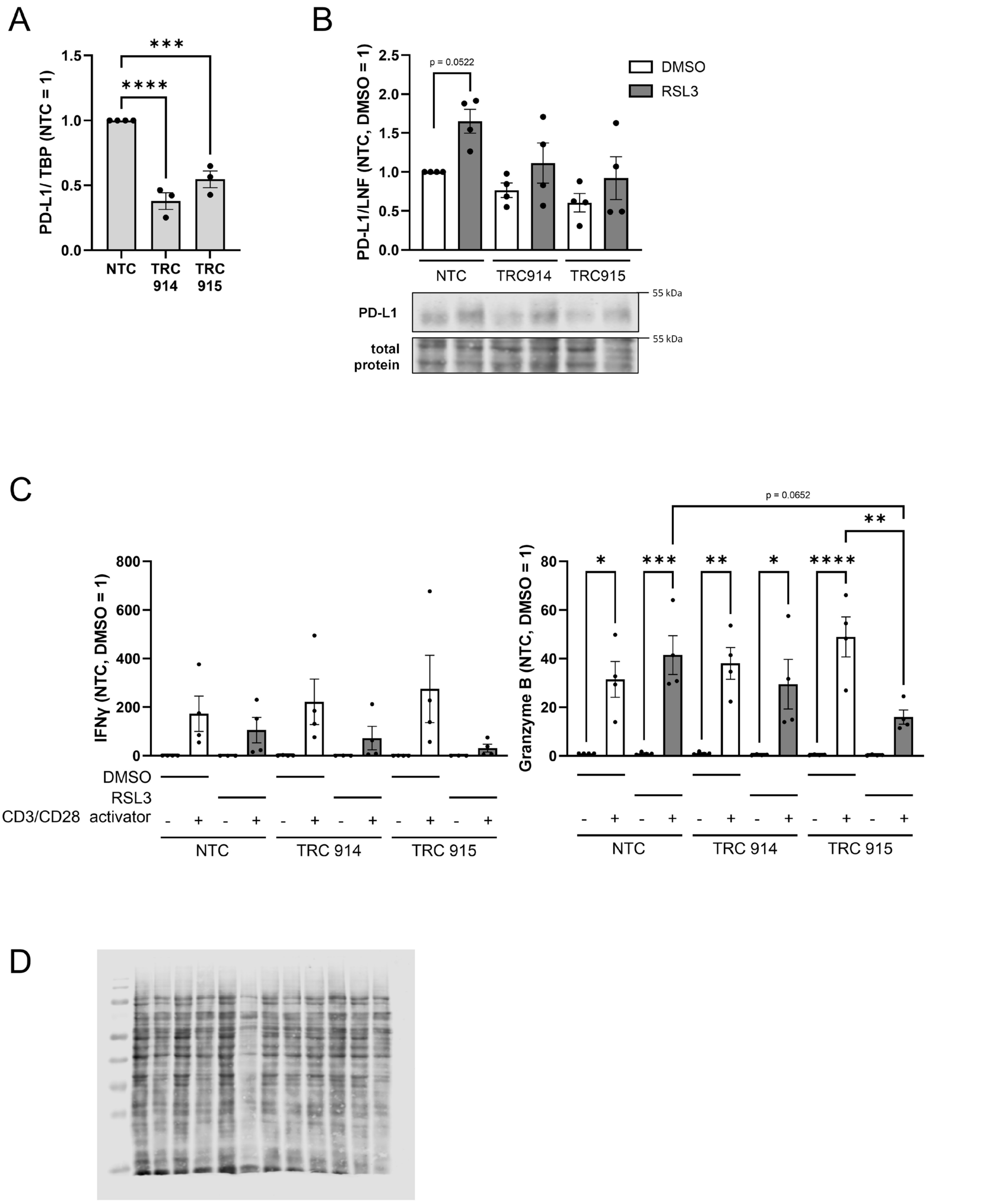

Figure S 4. Ferroptotic cells do not impede T cell activation via PD-L1.

Using shRNAs against PD-L1 and a non-targeted control (NTC) on LN229 glioblastoma cells, two stable knock-down cell lines, TRC914 and TRC915, were created. Knock-down cells were treated with 1 µM RSL3 and co-cultured with peripheral blood mononuclear cells (PBMCs) and T cell activation was measured by cytokine release. **A** PD-L1 mRNA in knock-down cells and NTC was measured and normalized to TBP. NTC was set to 1. Dunnett's multiple comparisons test was used. **B** Spheroid lysates of knock-down cells and NTC were analyzed for PD-L1 by Western analyzes. Data were normalized to lane normalization factor and NTC DMSO controls were set to 1. Šídák's multiple comparisons test was used. **C** Interferon gamma (IFNγ) and granzyme B levels were measured in the supernatants of PBMC and knockdown cell co-cultures after 2 days. Data from n = 4 individual donors. NTC treated with DMSO were set to 1. **D** Complete total protein stain for Western blots shown in Fig. S4 B. A Revert total protein stain was used, and membranes were scanned on an Odyssey CFx scanner.

Data are expressed as mean values ± SEM. *$p \leq 0.05$, **$p \leq 0.01$, ***$p \leq 0.001$, ****$p < 0.0001$; p values were calculated using ordinary one-way ANOVA and Tukey's multiple comparisons test if not stated otherwise.
